# *AVPR1a* and *SLC6A4* Gene Polymorphisms Are Associated with Creative Dance Performance

**DOI:** 10.1371/journal.pgen.0010042

**Published:** 2005-09-30

**Authors:** Rachel Bachner-Melman, Christian Dina, Ada H Zohar, Naama Constantini, Elad Lerer, Sarah Hoch, Sarah Sella, Lubov Nemanov, Inga Gritsenko, Pesach Lichtenberg, Roni Granot, Richard P Ebstein

**Affiliations:** 1 Department of Psychology, Mount Scopus, Hebrew University, Jerusalem, Israel; 2 Génétique Maladies Multifactorielles—Institut de Biologie de Lille, Lille, France; 3 Psychology, Behavioral Sciences, Ruppin Academic Center, Emek Hefer, Israel; 4 Israeli Olympic Medical Committee and Medical Faculty, Tel Aviv University, Te Aviv, Israel; 5 Sarah Herzog Memorial Hospital and Hebrew University, Jerusalem, Israel; 6 Musicology Department, Hebrew University, Jerusalem, Israel; University of Oxford, United Kingdom

## Abstract

Dancing, which is integrally related to music, likely has its origins close to the birth of *Homo sapiens,* and throughout our history, dancing has been universally practiced in all societies. We hypothesized that there are differences among individuals in aptitude, propensity, and need for dancing that may partially be based on differences in common genetic polymorphisms. Identifying such differences may lead to an understanding of the neurobiological basis of one of mankind's most universal and appealing behavioral traits—dancing. In the current study, 85 current performing dancers and their parents were genotyped for the serotonin transporter (*SLC6A4:* promoter region HTTLPR and intron 2 VNTR) and the arginine vasopressin receptor 1a *(AVPR1a:* promoter microsatellites *RS1* and *RS3)*. We also genotyped 91 competitive athletes and a group of nondancers/nonathletes (*n* = 872 subjects from 414 families). Dancers scored higher on the Tellegen Absorption Scale, a questionnaire that correlates positively with spirituality and altered states of consciousness, as well as the Reward Dependence factor in Cloninger's Tridimensional Personality Questionnaire, a measure of need for social contact and openness to communication. Highly significant differences in *AVPR1a* haplotype frequencies *(RS1* and *RS3),* especially when conditional on both *SLC6A4* polymorphisms (HTTLPR and VNTR), were observed between dancers and athletes using the UNPHASED program package (Cocaphase: likelihood ratio test [LRS] = 89.23, *p* = 0.000044). Similar results were obtained when dancers were compared to nondancers/nonathletes (Cocaphase: LRS = 92.76, *p* = 0.000024). These results were confirmed using a robust family-based test (Tdtphase: LRS = 46.64, *p* = 0.010). Association was also observed between Tellegen Absorption Scale scores and *AVPR1a* (Qtdtphase: global chi-square = 26.53, *p* = 0.047), *SLC6A4* haplotypes (Qtdtphase: chi-square = 2.363, *p* = 0.018), and *AVPR1a* conditional on *SCL6A4* (Tdtphase: LRS = 250.44, *p* = 0.011). Similarly, significant association was observed between Tridimensional Personality Questionnaire Reward Dependence scores and *AVPR1a RS1* (chi-square = 20.16, *p* = 0.01). Two-locus analysis (*RS1* and *RS3* conditional on HTTLPR and VNTR) was highly significant (LRS = 162.95, *p* = 0.001). Promoter repeat regions in the *AVPR1a* gene have been robustly demonstrated to play a role in molding a range of social behaviors in many vertebrates and, more recently, in humans. Additionally, serotonergic neurotransmission in some human studies appears to mediate human religious and spiritual experiences. We therefore hypothesize that the association between *AVPR1a* and *SLC6A4* reflects the social communication, courtship, and spiritual facets of the dancing phenotype rather than other aspects of this complex phenotype, such as sensorimotor integration.

## Synopsis

Dancing, integrally related to music, likely has its origins close to the birth of *Homo sapiens*. The authors hypothesized that there are differences in aptitude, propensity, and need for dancing that may be based on differences in common genetic polymorphisms. Identifying such differences may lead to an understanding of the neurobiological basis of dancing.

Variants of the serotonin transporter and the arginine vasopressin receptor 1a genes were examined in performing dancers, elite athletes, and nonathletes/nondancers. The serotonin transporter regulates the level of serotonin, a brain transmitter that contributes to spiritual experience. The vasopressin receptor has been shown in many animal studies to modulate social communication and affiliative behaviors. Notably, dancers scored high on the Tellegen Absorption Scale, a correlate of spirituality, and the Reward Dependence factor in Cloninger's Tridimensional Personality Questionnaire, a measure of empathy, social communication, and need for social contact. Significant differences were observed in allele frequencies for both genes when dancers were compared to athletes as well as to nondancers/nonathletes. These two genes were also associated with scores on the Tellegen Absorption Scale and Tridimensional Personality Questionnaire Reward Dependence, suggesting that the association between these genes and dance is mediated by personality factors reflecting the social communication, courtship, and spiritual facets of the dancing phenotype.

## Introduction

“With the creation of the universe, the dance too came into being, which signifies the union of the elements. The round dance of the stars, the constellation of planets in relation to the fixed stars, the beautiful order and harmony in all its movements, is a mirror of the original dance at the time of creation.”

Lucian of Samosata (~125 to 180 A.D.), *On Dance (De Saltatione)*

Dance, an art form closely allied to music, has been little studied from the neuroscience or genetic perspective, despite its significance in all cultures throughout the ages. Dance, like music, is an activity dating to prehistoric times that is sometimes a sacred ritual, sometimes a form of communication, and sometimes an important social and courtship activity; finally, dance is an art form that exists in every culture and manifests diverse paths [1]. Dance, as an expressive art form, is often considered inherently creative, especially when compared with a “nonartistic” domain. It is also a cultural form that results from creative processes that manipulate human bodies in space and time (“embodiment”). In many ways, dance is also a part of music, to which it is integrally related. Finally, professional dancers possess an exceptional talent, and as noted by Kalbfleisch [2], “Exceptional talent is the result of interactions between goal-directed behavior and nonvolitional perceptual processes in the brain that have yet to be fully characterized and understood by the fields of psychology and cognitive neuroscience.”

Dance may appear to be an unusual phenotype for human molecular genetics studies, but it is no more so than two closely related phenotypes, music [3] and athletic performance [4−6], that have both become subjects of molecular research. There is accumulating contemporary interest in the neuroscience of music [3,7−11] providing “proof of principle” that a widespread pursuit historically considered as part the human art and cultural heritage also has a solid basis in neuroscience, evolution, and genetics. Both music and athletic performance are complex phenotypes, the presentation of which is molded by environment and genes (and their interaction), especially in elite performers. A good example is absolute pitch, a relatively “clean” musical phenotype, of which the occurrence in approximately 20% of professional musicians is dependent not only on intrinsic ability but also on age of onset and intensity of musical training [11]. Similarly for athletic performance, evidence has accumulated over the past three decades for a strong genetic influence on human physical performance, with an emphasis on two sets of physical traits, cardiorespiratory and skeletal muscle function, that are particularly important for performance in a variety of sports [4]. A number of individual genetic variants associated with elite athletes have been provisionally identified, but there is little argument that elite athletes as well as elite musicians likely possess other characteristics related to personality and emotion that also contribute to their performance.

We suggest the notion that the “dance” phenotype is no more difficult to define than other complex human behavioral phenotypes (schizophrenia, attention deficit, personality, violence, and others) that have been shown to be both heritable and amenable to genetic analysis. Dancers fulfill a set of criteria with considerable face validity (similar in principle to the usual *Diagnostic and Statistical Manual of Mental Disorders*–style “symptom checklist” [12]) that both identifies and distinguishes one disorder from another. For example, the US Department of Labor suggests that the following qualities, inter alia, are required to be a professional dancer: flexibility, agility, coordination, grace, a sense of rhythm, a feeling for music, and a creative ability to express oneself through movement [13].

In our ongoing studies of the genetic basis of human personality [14−16], we have recruited currently performing dancers (*n* = 85) who train for at least 10 h per week, because we thought that a study of this group would help us understand why some individuals are endowed with creative and artistic abilities or inclinations. Toward this end, dancers were characterized using both psychosocial instruments and common genetic polymorphisms. Of particular interest are the Tridimensional Personality Questionnaire (TPQ) [17] and the Tellegen Absorption Scale (TAS) [18], which, respectively, measure aspects of social communication (TPQ Reward Dependence) and spirituality (TAS), personality facets important in the dance phenotype.

We investigated two polymorphic genes that we hypothesized to add to artistic creativity: the arginine vasopressin 1a receptor *(AVPR1a)* and the serotonin transporter *(SLC6A4).* The *SLC6A4* long promoter allele is more efficient at the level of transcript, producing more transporter protein that presumably more effectively removes serotonin from the synapse [19]. Both common intron 2 VNTR repeats (10 and 12) enhance transcription [20], although individual repeat elements differ in their activity in embryonic stem cell models [21]. In lower vertebrates, the promoter region repeat elements of the AVPR1a receptor determine brain-specific expression patterns and are responsible for differences in patterns of social communication across species [22]. In humans, the functional significance of the promoter repeats remains to be elucidated, although association between these repeats and social communication in humans was recently suggested [14,23,24].

We considered that *AVPR1a* might contribute to the dance phenotype, reflecting this gene's role in affiliative, social, and courtship behaviors [25], activities that are vital in many kinds of human dancing. Dancing also taps into human spiritual resources as evidenced by the role of dancing in sacred rituals [1]; it has been shown that serotonin plays a role in human spiritual experiences [26]. Additionally, use of ecstasy, a serotonergic neurotoxin, at rave dances and dance clubs [27] further links serotonin to both dancing and states of altered consciousness, two phenomena also linked in the absence of drugs. Finally, many studies show that serotonin enhances the release of vasopressin in the brain [28], suggesting the notion that these two genes, *AVPR1a* and *SLC6A4,* are also likely to exhibit epistasis, or gene−gene interactions, in association studies that reflect their interaction at the level of individual neurons as well as on the plane of neurotransmitter pathways. Interestingly, serotonin and vasopressin interact in the hypothalamus to control communicative behavior [29].

## Results

We first examined the arginine vasopressin 1a receptor *(AVPR1a)* promoter region microsatellites (allele frequencies for *RS1* and *RS3* are shown in Table 1) and the serotonin transporter gene *(SLC6A4),* initially using a case-control design by implementing the Cocaphase routine in the UNPHASED package to compare allele and haplotype frequencies between two groups, dancers versus athletes (Table 2). Comparing dancers with athletes is of interest because both groups of subjects demonstrate physical prowess and dedication to a demanding training routine but are expected to differ in musical aptitude and inclination. Indeed, dancers are sometimes considered performing athletes [30]. Single-locus analysis showed significance for the *RS3* marker and a two-locus haplotype *(RS1* and *RS3).* When the *AVPR1a* polymorphisms were analyzed conditional on the *SLC6A4* polymorphisms, highly significant differences in allele and haplotype frequencies were observed (Table 2). Similar results were obtained when dancers were compared to the nondancers/nonathletes (*AVPR1a RS1* and *RS3* conditional on *SLC6A4* HTTLPR and VNTR: likelihood ratio test [LRS] = 92.76, DF = 44, *p* = 0.00002).

**Table 1:**
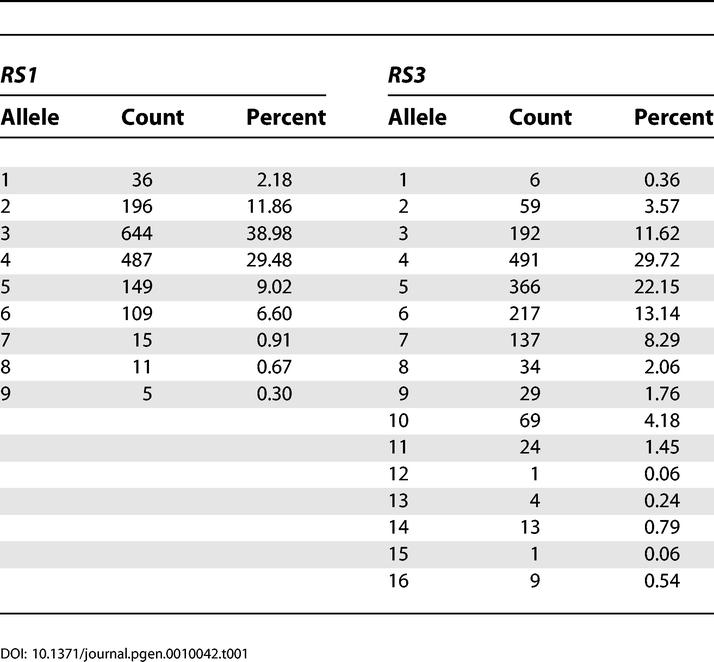
*AVPR1a* Allele Frequency for *RS1* and *RS3*

**Table 2:**
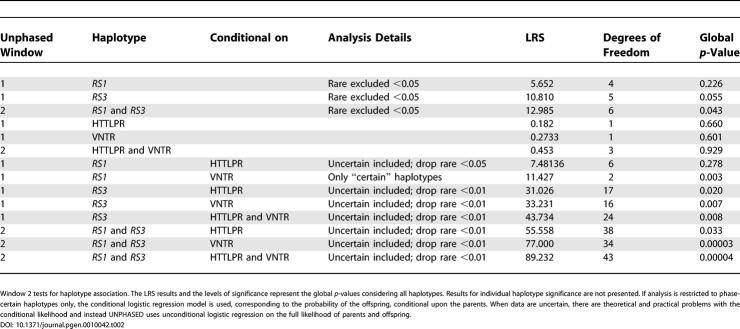
Case-Control Design (Cocaphase): Comparing Dancers to Athletes

We also tested preferential transmission of allele and haplotypes using a robust family-based design implemented in the Tdtphase routing of UNPHASED by assigning “dancer” as an “affective” status (Table 3). Preferential transmission of the *AVPR1a* microsatellite alleles from heterozygous parents to their dancer offspring was observed both for individual genes and haplotypes. The most significant evidence for transmission was observed when *RS1* and/or *RS3* transmission was conditional on *SLC6A4* HTTLPR and/or VNTR polymorphisms.

**Table 3:**
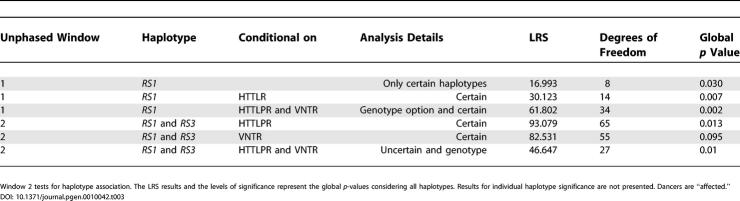
Testing Association between *AVPR1a* and *SLC6A4* and Dancing in a Family-Based Design (Tdtphase)

**Figure 1:**
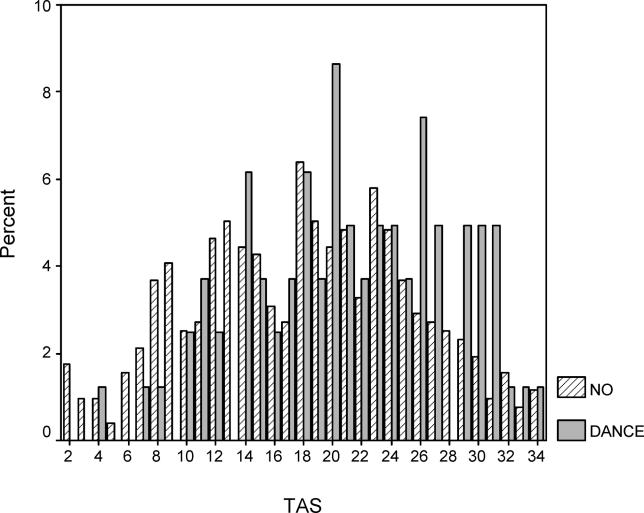
Distribution of TAS in Female Dancers and Nondancers/Nonathletes

**Figure 2:**
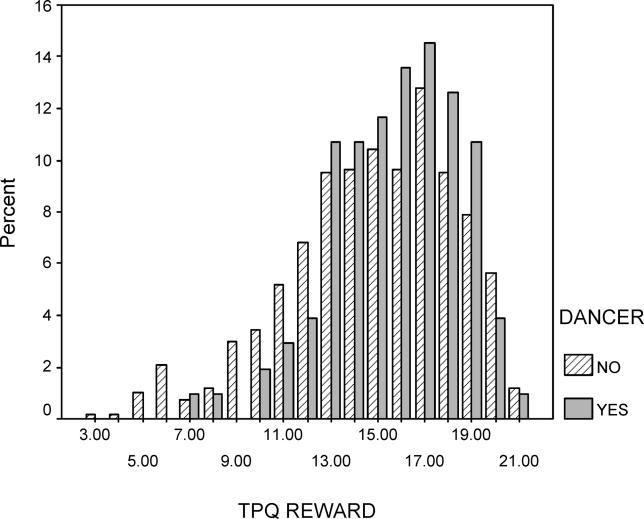
Distribution of TPQ Reward Dependence Scores in Female Dancers and Nondancers/Nonathletes

To better understand the psychobiological mechanism by which the *AVPR1a* gene contributes to the “dancer” phenotype, we also compared dancers to nondancers on several psychosocial scales (Table 4). Highly significant differences between dancers and athletes are observed for two variables: the TAS [18] (effect size = 0.72 standard deviation [SD] units) and TPQ Reward Dependence [17] (effect size = 0.68 SD units). Less significant differences were observed for TPQ Self-Esteem [31], Fear of Failure [32], and Drive for Success [32]. Absorption is a tendency to experience episodes of “total” attention that fully engage perceptual, enactive, imaginative, and ideational resources [18]. The high scores of the dancers on the TAS and TPQ Reward Dependence suggested it would be worthwhile to examine association between these scores and both genes in the full group (nondancers and nonathletes) of subjects (*n* = 872 from 414 families) who we recruited in our personality studies. As shown in Table 5 (see Figures 1 and 2 for the distribution of TAS and TPQ Reward Dependence scores in dancers versus nondancers), using the family-based design there is an association between the *AVPR1a* and *SLC6A4* genes and scores on the TAS. The most common (42%) *SLC6A4* haplotype, short HTTLPR promoter region-12 repeat VNTR repeat, shows the strongest association with high TAS scores (*p* = 0.018). The two-locus *RS1* and *RS3* haplotype is also significantly associated with the TAS (*p* = 0.047). We also categorized TAS scores (“affected” as top 20%; score > 24, *n* = 203 affected) and observed significant association when *RS1* and *RS3* were conditional on the HTTLPR and VNTR polymorphisms (*p* = 0.01). As shown in Table 6, association was also observed between the *AVPR1a RS1* microsatellite and TPQ Reward Dependence (*p* = 0.009), and the results were highly significant (*p* = 0.00097) for two-locus analysis when both *RS1* and *RS3* microsatellites were conditional on both *SCL6A4* polymorphisms (*n* = 181 “affected” top 20% scores > 18). No association was observed between these two genes and TPQ Self-Esteem, Drive for Success, or Fear of Failure (data not shown).

**Table 4:**
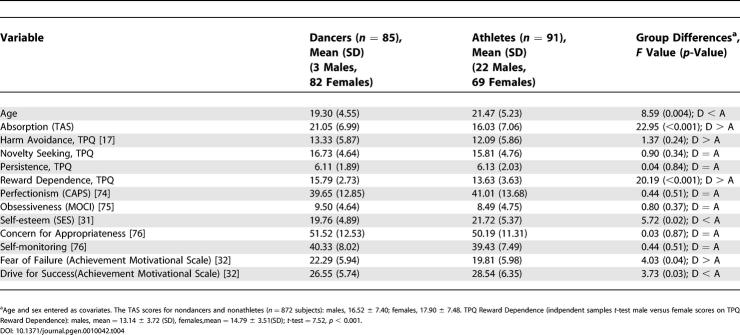
Comparison between Dancers and Athletes on Demographic and Psychosocial Scales

**Table 5:**
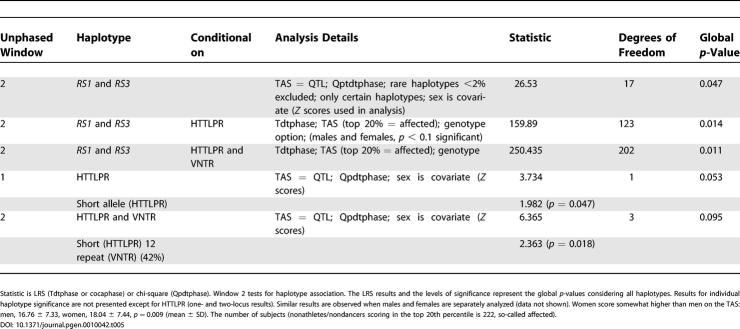
Family-Based Design (TDT) and Testing Association between *AVPR1a*, *SLC6A4,* and TAS

**Table 6:**
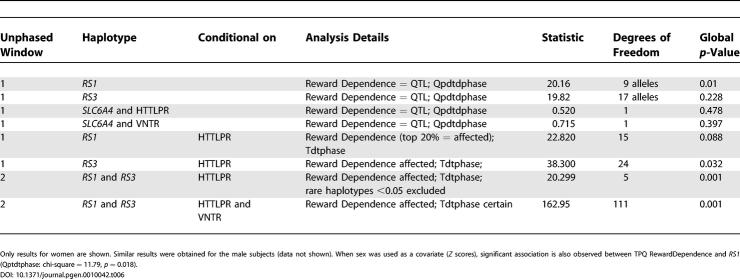
Family-Based Design (TDT) and Testing Association between *AVPR1a*, *SLC6A4,* and TPQ Reward Dependence

We also examined other polymorphic genes of some interest in neuroscience but failed to show a significant association with creative dancing. These included the dopamine D5 microsatellite marker *(DRD5),* linked in some studies to attention deficit [33,34], the insulin-like growth factor 2 (*IGF2*, three single nucleotide polymorphisms), linked to eating disorders and self-report measures of altruism [15,35], catechol-*O*-methyltransferase *(COMT)* [36], linked to cognitive function and schizophrenia [37], and monoamine oxidase A *(MAOA)* [38], linked to violence [39]. Preliminary evidence for an association between the dopamine D4 receptor *(DRD4)* [35,40] and the dancing phenotype was observed, and further studies of this gene are now in progress.

## Discussion

Few, if any, genes have been associated with artistic creativity, and none, to our knowledge, specifically with dancing. The current study considered two genes, *AVPR1a* and *SCL6A4,* that are significantly associated with performing dancers. Although the association seems robust and is significant both by case-control and family-based designs, it is nevertheless a challenge to unravel the brain mechanisms by which these genes partially contribute to dancing, a phenotype extending across musical processing, motor coordination, and artistic creativity.

The association between *AVPR1a* and *SLC6A4* polymorphisms and creative dancing does not exclude the presence of the same polymorphisms in nondancing groups of subjects. Almost all of us dance and almost all of us have engaged in sports. What the current study suggests is that the combination of polymorphic variants contributing to creative dancing is overrepresented in the dancers. There is no reason to suggest that the nondancer athletes or the control group of nondancers/nonathletes are devoid of these polymorphisms, but the current study provides evidence that these variants are relatively scarce in other groups not specifically selected for the creative dancing phenotype. Importantly, we not only compared creative dancers to performing athletes but also validated the case-control design using a family-based study that avoids the conundrum of a comparison control group that might be “contaminated” with polymorphisms contributing to creative dancing. As for most complex traits, the effect size of these two genes is small and in Risch's terminology will have small displacement [41]

The *AVPR1a* gene makes a profound contribution to affiliative and social behavior in lower vertebrates [25]. It is hypothesized that microsatellite promoter-region instability may be a major factor producing diversity in both region-specific gene expression and the resulting phenotypes [42]. In humans, however, the role of the *AVPR1a* repeat regions has yet to be resolved. Nevertheless, despite the dearth of research regarding the molecular mechanisms involved, the *AVPR1a* promoter-region microsatellites have recently been associated with autism [23,24], a disorder whose core symptom is a deficit in social communication. Additionally, we have shown that this gene is associated with measures of social behavior in control subjects [14]. We suggest the notion that the association between *AVPR1a* and dancing may be reflecting the importance of social relations and communication in the dance form and that both dance and its associated gene, *AVPR1a,* contribute to molding social interactions from the molecular level to the dance floor. As noted by Kaeppler [1], the cultural form produced in dance, although transient, has structured content and is a visual manifestation of social relations that may be the subject of an elaborate aesthetic system. It seems likely that one of the many prerequisites for a successful dance career is the capacity for social communication through dance (“embodiment”).

Intriguingly, Darwin recorded in *The Voyage of the Beagle* (Chapter 19) his Australian encounter with the so-called White Cockatoo” aboriginal men and their performance in a *corrobery,* or “great dancing-party.” As Darwin notes, “Perhaps these dances originally represented actions, such as wars and victories; there was one called the Emu dance, in which each man extended his arm in a bent manner, like the neck of that bird. In another dance, one man imitated the movements of a kangaroo grazing in the woods, whilst a second crawled up, and pretended to spear him” [43]. It is worth noting in this context that the native Australians arrived on that continent approximately 50,000 y ago [44]. Similarly, Native American, also known for their complex dance culture [1], are estimated to have arrived in North America about 20,000 to 15,000 calendar years before the present [45]. We conjecture that both groups arrived on their respective continents already with a dance culture (alternatively there was parallel “cultural” evolution of this art form) that we speculate is likely to have originated before the African exodus. The earliest evidence for dance is derived from a cave painting in Creswell, England, that depicts dancing women and is dated approximately 13,000 y ago [46]. We hypothesize that this early evidence for dancing and its occurrence in groups geographically separated by thousands of years during our prehistory suggests a genetic basis for this behavior in *H. sapiens.*

Another aspect of dance is its spiritual side (e.g., sacred dancing across many diverse cultures) and the relationship of dance to altered states of consciousness; for example, in the Korean Salpuri dance, an ecstatic trance state is induced that results in changes in alpha wave activity [47]. Dances in which the participant (often a woman) enters into a trance and may lose consciousness have been used in a therapeutic setting (e.g., to dispossess “demons”) in diverse cultures, including a North African Jewish community [48]. We suggest the notion that the association we observe between *SLC6A4* and dance is perhaps related to the need for altered consciousness states that subjects participating in and performing this art form sometimes have. Dancing may have its origins in shamanism, which sometimes used a potent and synergetic mix of music and dancing (and sometimes drugs) to alter consciousness [49]. Perhaps a prerequisite for some types of dancing, in both sacred and more modern “profane” versions as either an artistic performer or a participant, is the ability to enter into such a higher state of awareness.

The personality construct of absorption is generally measured using the TAS, a self-report personality instrument with good psychometric properties [50]. The TAS has been widely applied in research in an attempt to evaluate the significance of an individual's ability to attend intensely and imaginatively to stimuli. It has been found to correlate positively with spirituality and altered states of consciousness, such as an intrinsic religious orientation involving the internalization of religious tenets so they provide meaning and direction [51], and the frequency and type of reported spontaneous mystical, visionary, and paranormal experiences [18,50,52–55].

The short *SLA6A4* promoter region polymorphism that we find associated with scores on the TAS characterizes a less efficient promoter, and in subjects carrying the short allele, less transporter protein is synthesized [19,56], which would lead to altered synaptic levels of serotonin. It is not surprising therefore that we observe an association between TAS scores (indicating in some individuals a propensity to have mystical, visionary experiences, and/or “artistic” sensitivity) and a common genetic polymorphism that modulates synaptic serotonin levels. A large body of evidence links hallucinogens, drugs that alter consciousness, to serotonin, and it is thought that hallucinogens stimulate 5-HT_2A_ receptors, especially those expressed on neocortical pyramidal cells [57]. Altered serotonin levels in carriers of the SLC6A4 promoter region allele might predispose such individuals to a greater ability for imagery and attention to stimuli (especially to musical stimuli) that we hypothesize may provide part of the “hard wiring” that talented and devoted individuals need to perform in an art form that combines a unique combination of both musical and physical skills.

Individuals high in TPQ Reward Dependence tend to be tender-hearted, loving and warm, sensitive, dedicated, dependent, and sociable [58]. They seek social contact and are open to communication with other people. Typically, they find people they like everywhere they go and are sensitive to social cues, which facilitates warm social relations and understanding of others' feelings. The observed association between TPQ Reward Dependence scores and *AVPR1a* is consistent with the role of the arginine vasopressin receptor in social communication as demonstrated in extensive animal experiments [59]. The association between *AVPR1a* and Reward Dependence personality traits strengthens the notion that this gene contributes to dancing through its contribution to social communication.

**Figure 3:**
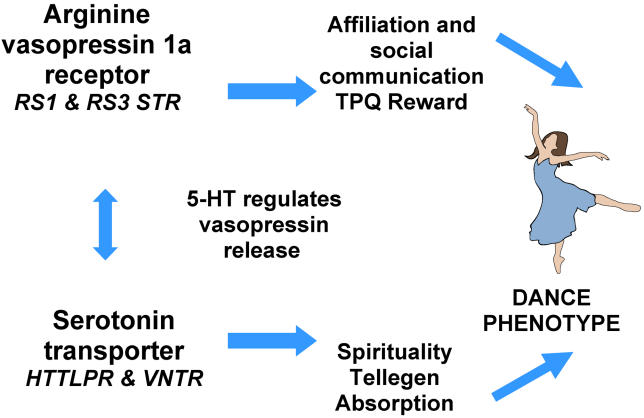
Epistatic Interaction between *AVPR1a* and *SLC6A4* Contributes to the Creative Dance Phenotype Promoter region polymorphisms in the AVPR1a receptor region possibly contribute to regional differences in brain arginine receptor 1a expression patterns [42]. Vasopressin release, and subsequent AVPR1a receptor activation, is partially regulated by serotonin (5-HT) [28]. 5-HT is removed from the synapse by the serotonin transporter *(SLC6A4),* which plays a major role in regulation of synaptic levels of this neurotransmitter. In turn, synaptic *SLC6A4* mRNA and protein levels are controlled in part by the presence or absence of a promoter region 44-bp insertion/deletion [19]. Subjects with polymorphic variants of these two genes are therefore predicted to show differences in serotonergic and vasopressin tone that contribute to differences in higher psychological constructs including TPQ Reward Dependence (associated with *AVPR1a* and *AVPR1a* × *SLC6A4* gene × gene interaction) and TAS (associated with *SLC6A4* and *AVPR1a* × *SLC6A4* gene × gene interaction). Dancers score high on these two personality constructs, suggesting the hypothesis that the association between *AVPR1a* and *SLC6A4* polymorphisms and dancing is likely mediated by the action of these two genes primarily on social communication (measured by TPQ Reward Dependence scores) and spirituality (measured by TAS scores). Similar to genes contributing to other complex traits, there are no “dancing” genes but rather common polymorphisms that contribute to simpler endophenotypes [77], such as TPQ Reward Dependence and TAS, that constitute some of the critical psychological underpinnings of the dance phenotype.

Figure 3 summarizes our notions of how polymorphisms in the *AVPR1a* and *SLC6A4* genes contribute to the dance phenotype.

The proposed role of *AVPR1a* in contributing to the dance phenotype in humans as provisionally shown in the current report has a solid evolutionary basis. Across the vertebrates, vasopressin plays a key role in courtship behavior that frequently involves elements of song and dance. For example, male zebra finches *(Taeniopygia guttata)* sing directed song to females as an integral part of a courtship display that also includes elements of dance [60]. The choreography of the dance presumably conveys or enhances some part of the message that is carried by the individual's learned song, although the exact importance and function of the dance are not known. Furthermore, arginine vasopressin plays a key role courtship behavior in zebra finches [61] as well as in other bird species such as the territorial field sparrow *(Spizella pusilla)* [62], as it does in social behaviors in mammals and other vertebrates [22]. There is also evidence in humans that vasopressin is important in maternal and romantic love. Imaging studies have shown that brain areas rich in vasopressin receptors are activated when subjects are shown pictures designed to evoke feelings of attachment [63]. The observation discussed above that *AVPR1a* is associated with a temperament trait, Reward Dependence, also strengthens the conjectured role of this gene in human social communication. Thus, studies in humans as well as in many other animal species suggest to us the reasonable notion that variations in *AVPR1a* microsatellite structure might also predispose some individuals to excel in dancing. Darwin observed that vocalization is a form of emotional expression, but among neuroethologists, questions concerning the role of emotion in vocal (and dance) communication have been superseded by questions that concern sensorimotor integration [64]. We believe that the two genes we have identified with dancing in humans are likely involved in the emotional side of dance rather than in the sensorimotor mechanics of this complex phenotype. To quote West and King [65], “Animals do not perceive or communicate for the sake of perceiving or producing a display, but for the sake of managing a social environment.” In this context, it is easier to see how the *AVPR1a* receptor microsatellite polymorphisms contribute to human dance. Human dancing can be understood in part as a form of courtship and social communication that shares a surprisingly conserved evolutionary history, characterized by apparently common neurochemical and genetic mechanisms, with mating displays and affiliative behavior observed across the vertebrates.

## Materials and Methods

### Dancers.

One hundred seven dancers, who trained for a minimum of 10 h per week and perform regularly, were initially recruited for this study from professional dance classes and dance companies in Israel. From this group, DNA was obtained from 85 dancers and their parents and was successfully genotyped (82 females and three males). The main types of dance performed by the 85 dancers who were genotyped were classical ballet, modern or contemporary dance, and jazz ballet. The average age of the dancers was 19.30 ± 4.55 y (SD).

This project was approved by the Herzog Hospital Helsinki Committee and the Israeli Ministry of Health, Genetics Section, and all subjects gave informed consent.

### Athletes.

Ninety-one (22 males and 69 females) competitive (nonperforming) athletes (and their parents) were recruited. The athletes competed regularly at the highest echelons of their respective sports in Israel and often abroad; 36 (32 females and four males) were endurance athletes, mostly runners and swimmers; 39 (24 females and 15 males) competed at a high level in ballgames such as basketball and volleyball; nine (eight females and one male) competed in technical sports such as sailing and fencing; and seven (five females and two males) competed in the martial arts. Sixty-seven female nonperforming athletes were recruited via sports unions and the Israeli National Sports Institute. Their average age was 21.47 ± 5.23 y.

### Nonathletes/nondancers.

This group (*n* = 872 from 414 families) has been described in our previous studies [14,15]. They were primarily university students recruited from the Hebrew University campus, Mt. Scopus, Jerusalem. The average age of the subjects was 21.44 ± 4.37 y (range, 13−36 y).

### DNA extraction and genotyping.

DNA was obtained from all family members and extracted with use of the MasterPure kit (Epicentre, Madison, Wisconsin, United States). Amplification of the *RS1* and *RS3* arginine vasopressin 1a microsatellites *(AVPR1a)* was achieved using the following pair of primers: *RS1* [24,66] forward (fluorescent) 5′-AGG GAC TGG TTC TAC AAT CTG C-3′ and reverse 5′-ACC TCT CAA GTT ATG TTG GTG G-3′; *RS3* [24,66] forward (fluorescent) 5′-CCT GTA GAG ATG TAA GTG CT-3′ and reverse 5′-TCT GGA AGA GAC TTA GAT GG-3′. Microsatellite haplotypes *(RS1* and *RS3)* showed mild linkage disequilibrium (UNPHASED: global D′ = 0.339). The allele frequencies for *RS1* and *RS3* are shown in Table 1.

Each reaction mixture contained 0.5 μM primer and 20 ng of DNA. A ReddyMix master mix (Thermoprime plus DNA polymerase) was used (Abgene, Surrey, United Kingdom) at a magnesium concentration of 1.5−2.5 mM MgCl_2_. ReddyMix buffer consisted of 75 mM Tris-HCl (pH 8.8 at 25 °C), 20 mM (NH_4_)_2_SO_4_, and 0.01% (v/v) Tween 20. The sample was initially heated at 95 °C for 5 min followed by 30 cycles of 95 °C (30 s), 55 °C (30 s), and 72 °C (40 s), and a final extension step of 72 °C for 10 min. The PCR product was analyzed on an ABI 310 DNA analyzer (Applied Biosystems, Foster City, California, United States).

A subroutine of Merlin was used to test for conformity with Hardy–Weinberg equilibrium (HWE) and no departure from HWE was observed for either microsatellite.

### 5-HTTLPR.

PCR amplification was carried out using a ReddyMix kit (Abgene). The primers used were forward 5′-GGCGTTGCCGCTCTGAATGC-3′ and reverse 5′-GAGGGACTGAGCTGGACAACC-3′. The reaction mixture contained the following components: 0.5 μM primers, 20 ng of DNA, and 5% DMSO in a total volume of 10 μl. ReddyMix buffer consisted of 75 mM Tris-HCl (pH 8.8 at 25 °C), 20 mM (NH_4_)_2_SO_4_, and 0.01% (v/v) Tween 20. After an initial denaturation step of 94 °C for 5 min, amplification was carried out for 35 cycles (94 °C for 30 s, 55 °C for 30 s, and 72 °C for 90 s) in a PerkinElmer (Wellesley, California, United States) Cetus 9600 thermal cycler. A 5-min final extension at 72 °C was used. The reaction mixture was electrophoresed on a 3% agarose gel (Ameresco, Solon, Ohio, United States) with ethidium bromide to screen for genotypes.

### VNTR.

PCR amplification was carried out for the intron 2 VNTR with the following primers: forward 5′- TCAGTATCACAGGCTGCGAG-3′ and reverse 5′-TGTTCCTAGTCTTACGCCAGTG-3′ [67]. The reaction mixture contained the following components: 0.5 μM primers, 20 ng of DNA, and 5% DMSO in a total volume of 10 μl. ReddyMix buffer consisted of 75 mM Tris-HCl (pH 8.8 at 25 °C), 20 mM (NH_4_)_2_SO_4_, and 0.01% (v/v) Tween 20. After an initial denaturation step of 94 °C for 5 min, amplification was carried out for 35 cycles (94 °C for 30 s, 61 °C for 30 s, and 72 °C for 30 s) in a PerkinElmer Cetus 9600 thermal cycler. A 10-min final extension at 72 °C was used.

The SLC6A4 repeats were in LD: global D′ = 0.71 (UNPHASED). There was no deviation from HWE. The allele frequency of the HTTLPR polymorphism is 50.4% long (plus 44-bp insertion) and 49.4% short (44-bp deletion); there were four individuals with a rare short allele [68] who were excluded from the analyses. The allele frequency of the VNTR polymorphism is 27.6% of the 10 repeat, 72.2% of the 12 repeat, and four individuals with a 9 repeat who were excluded from the genetic analyses.

The percentage of heterozygote parents is as follows: *AVPR1a RS1* = 72%, *RS3* = 82%; *SLC6A4* HTTLPR = 58%, VNTR = 44%.

### Quality of genotyping.

Quality of genotyping was determined as follows. (1) All families were initially screened for Mendelian consistency using seven highly polymorphic microsatellite markers. Problematic families who were not consistent with Mendelian inheritance (<1%) were excluded from the study. (2) Because all subjects in the current study were family members, genotype errors with SNPs appearing as Mendelian inconsistency, and automatically flagged by the statistical program, were reexamined either for data entry errors or by regenotyping such families for the aberrant SNPs. (3) In all cases of borderline classifications, when reading the ABI output, the PCR procedure was repeated. (4) Quality control and estimation of error rate (percent of miscalled genotypes) were evaluated by reanalysis of 5% of the families. The observed error rate is estimated to be less than 0.5%. (5) Because deviation from HWE in random samples may be indicative of problematic assays [69], the frequency of the alleles was examined for HWE. Additionally, the genotype and allele frequencies were compared with published results from other investigations, and were found to be similar.

### Statistical methods.

We used the logistic-based variant of the transmission disequilibrium test (TDT), so-called ETDT [70], to assess association (and linkage) without confounding effect of population stratification. The TDT, in its simplest version, compares, for one allele, the number of times this allele is transmitted to the number of times where this allele is not transmitted to an affected offspring. Note that only heterozygous parents are informative. This approach can be extended to haplotypes.

The various tests we used are implemented in the program UNPHASED (http://www.rfcgr.mrc.ac.uk/~fdudbrid/software/unphased/). UNPHASED [71] is a suite of programs for association analysis of multilocus haplotypes from unphased genotype data. UNPHASED currently includes the following programs: UNPHASED, which is the graphical front end to the analysis programs; Tdtphase for TDT and HHRR analysis for nuclear families; Cocaphase, for case-control data; Qtphase, for quantitative traits in unrelateds; Pdtphase, for pedigree disequilibrium tests; and Qpdtphase, for quantitative trait pedigree disequilibrium tests.

### Conditional analysis.

Here, the ETDT is adapted to test for an effect at a secondary locus or marker conditional on the association of a candidate disease locus in case–parent triads. Considering gametic haplotypes of a candidate (or established) disease locus and a neutral marker, it is expected that haplotypes with identical alleles at the candidate disease locus, but different alleles at the marker, have equal transmission probabilities. ETDT transmission probabilities can be estimated and, in this adaptation, can be tested for equality using a LRT called Conditional ETDT [72]. A significant difference in transmission of haplotypes identical at the candidate locus, but different at the secondary locus, provides evidence for the involvement of either the secondary locus or a locus in linkage disequilibrium with it.

Because some markers are microsatellites, with *RS1* = 9 and *RS3* = 17 alleles, including some rare alleles, we used a permutation procedure to confirm the asymptotic *p*-value. This procedure randomly permutes for parental alleles or haplotypes and the transmission and nontransmission status and computes the observed ETDT statistic. The *p*-value is the number of times a specific observed statistic is observed in *n* permutations divided by the number of permutations *(n)*.

For single-locus and haplotype analysis, UNPHASED calculates overall global *p*-values that consider multiple testing of haplotypes. Those values are included in all of the tables (“Global *p*-Values”).

However, regarding the more complicated problem of how to correct for multiple testing in association studies when there are potentially approximately 30,000 genes in the human genome, the reader is referred to the insightful article by Neale and Sham that discusses this problem [73]. In the current study, the *p*-values are nominal and not corrected for multiple testing.

## Abbreviations

HWE: Hardy–Weinberg equilibrium
LRS: likelihood ratio test
SD: standard deviation
TAS: Tellegen Absorption Scale
TDT: transmission disequilibrium test
TPQ: Tridimensional Personality Questionnaire
