# Correction: *AVPR1a* and *SLC6A4* Gene Polymorphisms Are Associated with Creative Dance Performance

**DOI:** 10.1371/journal.pgen.1008135

**Published:** 2019-04-26

**Authors:** Rachel Bachner-Melman, Christian Dina, Ada H Zohar, Naama Constantini, Elad Lerer, Sarah Hoch, Sara Sella, Lubov Nemanov, Inga Gritsenko, Pesach Lichtenberg, Roni Granot, Richard P Ebstein

The seventh author's name is spelled incorrectly. The correct name is: Sara Sella.
